# Development of metal adaptation in a tropical marine zooplankton

**DOI:** 10.1038/s41598-020-67096-1

**Published:** 2020-06-23

**Authors:** Khuong V. Dinh, Hanh T. Dinh, Hong T. Pham, Henriette Selck, Kiem N. Truong

**Affiliations:** 1 School of Biological Sciences, Washington State University, Pullman, WA USA; 2 Department of Science and Environment, Roskilde University, Universitetsvej 1, 4000 Roskilde, Denmark; 3 Cam Ranh Centre for Tropical Marine Research and Aquaculture, Institute of Aquaculture, Nha Trang University, No 2 Nguyen Dinh Chieu Street, Nha Trang City, Vietnam; 4 Northern National Broodstock Center for Mariculture, Research Institute for Aquaculture No 1, Xuan Dam Commune, Cat Ba, Hai Phong Vietnam; 5 Department of Environmental Engineering, Thuyloi University, 175 Tay Son, Dong Da, Hanoi Vietnam; 6 Department of Ecology, University of Science, Vietnam National University, Hanoi, 334 Nguyen Trai, Thanh Xuan, Ha Noi Vietnam

**Keywords:** Tropical ecology, Environmental impact

## Abstract

Tropical marine ecosystems are highly vulnerable to pollution and climate change. It is relatively unknown how tropical species may develop an increased tolerance to these stressors and the cost of adaptations. We addressed these issues by exposing a keystone tropical marine copepod, *Pseudodiaptomus annandalei*, to copper (Cu) for 7 generations (F1–F7) during three treatments: control, Cu and pCu (the recovery treatment). In F7, we tested the “contaminant-induced climate change sensitivity” hypothesis (TICS) by exposing copepods to Cu and extreme temperature. We tracked fitness and productivity of all generations. In F1, Cu did not affect survival and grazing but decreased nauplii production. In F2-F4, male survival, grazing, and nauplii production were lower in Cu, but recovered in pCu, indicating transgenerational plasticity. Strikingly, in F5-F6 nauplii production of Cu-exposed females increased, and did not recover in pCu. The earlier result suggests an increased Cu tolerance while the latter result revealed its cost. In F7, extreme temperature resulted in more pronounced reductions in grazing, and nauplii production of Cu or pCu than in control, supporting TICS. The results suggest that widespread pollution in tropical regions may result in high vulnerability of species in these regions to climate change.

## Introduction

The tropical coastal marine ecosystem in the South China Sea (SCS) is one of the most polluted regions worldwide^1–3^. High levels of metals have recently been reported in the coastal water of this region^4,5^, and metal pollution was one of the major causes of a recent massive fish kill in the SCS^6^. Furthermore, metals are persistent in the environment, and chronic exposure to metals often lasts beyond the course of one generation. Species may respond to multigenerational exposure to metals by evolving in phenotypic plasticity or development of adaptation. The role of phenotypic plasticity and adaptations in shaping the vulnerability of species to metals has been largely ignored in model predictions of ecotoxicological studies and ecological risk assessments of contaminants. Detangling phenotypic plasticity and adaptation is of crucial importance to understand how organisms may persist in nature and to improve ecological risk assessments, especially in the Anthropocene, where no coastal and marine ecosystem is free from anthropogenic disturbance^1^. A simple, yet powerful method to separate phenotypic plasticity and adaptation includes an experimental setup where organisms first are exposed and thereafter placed in clean conditions to examine if the stress response remains (adaptation) or recovers to control level (plasticity)^7^.

In general, organisms typically develop increased tolerance to long-term exposures to contaminants as a result of transgenerational plasticity, genetic adaptation, or both^8–11^. Multiple generational experiments have been applied to test how aquatic animals develop tolerance to stressors, such as metals^11^, polycyclic aromatic hydrocarbons^10^, pesticides^12^, algal toxins^13^, and temperature^14,15^. However, it remains a major challenge to detangle whether phenotypic changes in response to stressors are caused by transgenerational plasticity or from genetic adaptation. Adaptations to one stressor often come at the cost of reduced plasticity^16^ or reduced capacity to deal with additional stressors^17^. There is increasing evidence that transgenerational plasticity and adaptations to non-contaminant stressors may alter the sensitivity of organisms to a range of contaminants^12,18–21^. For example, Dinh *et al*.^22^, using space-for-time substitution, showed that thermal adaptation of aquatic insects results in a higher vulnerability to pesticides. This result supports the “climate-induced toxicant sensitivity” hypothesis^23^. However, the “contaminant-induced climate change sensitivity” hypothesis (TICS)^17,23^ remains to be tested.

This study aims to experimentally detangle phenotypic plasticity and adaptation as well as the cost of metal adaptation of a keystone tropical marine zooplankton species in response to exposure to a widespread contaminant (Cu) using an evolutionary experiment. We hypothesize that (1) tropical zooplankton develop adaptation to Cu after multigenerational exposure, and (2) the adaptation to Cu makes them more vulnerable to other stressors, particularly warming as predicted by Moe *et al*.^17^. To test these hypotheses, the tropical calanoid copepod, *Pseudodiaptomus annandalei*, was exposed to Cu (15 µg L^−1^) for seven generations. We quantified the changes in four key fitness traits, including survival, size at maturity, grazing rate (via faecal pellet production) and reproductive success (via nauplii production). A common tropical coastal copepod *P. annandalei* was chosen as the study organism as it plays an important role as the primary grazer on phytoplankton and small protozoans^24,25^. *P. annandalei* also provides the major food sources for marine fish larvae and juveniles in coastal marine ecosystems in SCS^26^.

## Results

### F1 generation

All females survived after 7 days, and survival of males was 72 ± 5% and 76 ± 5% (means ± SEs, n = 5) in control and the Cu treatment, respectively, and did not differ between the two treatments (F_1, 8_ = 0.20, *P* = 0.67). Likewise, size at maturity of females showed no statistical significance between the control and Cu treatment, and was 851 ± 8 µm and 864 ± 5 µm (means ± SEs, n = 5), respectively. Faecal pellet production did not differ between Cu and the control treatment (F_1, 8_ = 0.15, *P* = 0.71, Fig. 2A). Exposure to Cu reduced nauplii production by 19% compared to the control (F_1, 8_ = 33.97, *P* < 0.001, Fig. 2B).

**Figure 1:**
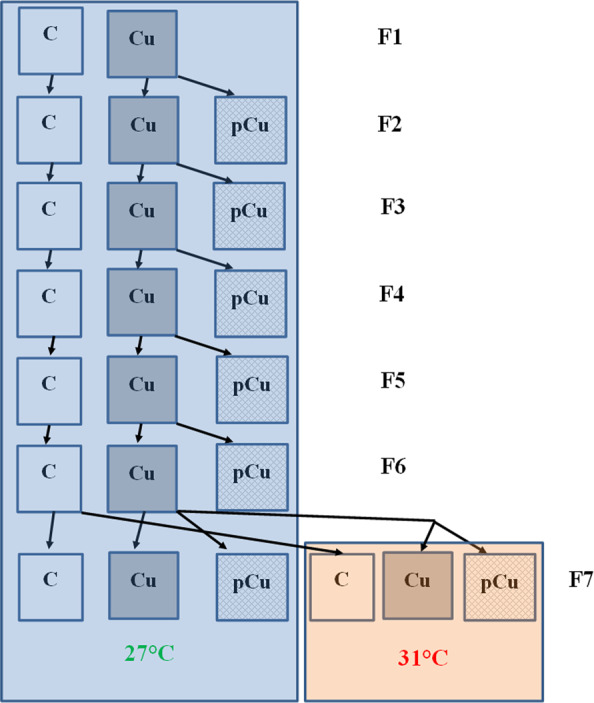
Experimental design diagram for testing the transgenerational plasticity and evolution of Cu tolerance of the tropical copepod *Pseudodiaptomus annandalei*. Cu: copper treatment: copepods were directly exposed to Cu; pCu: Cu pre-exposure treatment: offspring from Cu-exposed parents were returned to the control condition. F1-F7 refers to generations 1 to 7, respectively.

**Figure 2:**
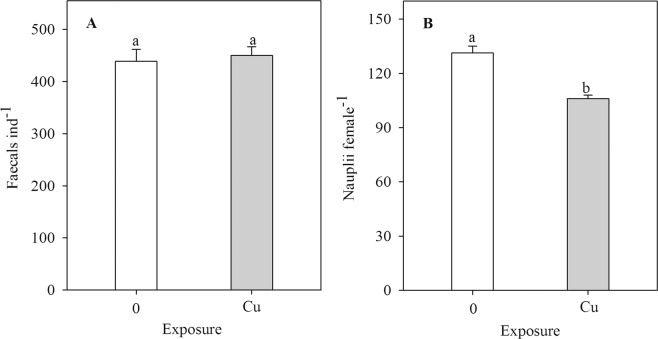
Faecal pellet (**A**) and nauplii production (**B**) of the F1 generation of *Pseudodiaptomus annandalei* as a function of Cu treatment. Data are visualized as mean + 1 SE. Different letters above the bars indicate significant differences among control and Cu (Duncan Posthoc tests).

### F2 – F6 generations

**Figure 3:**
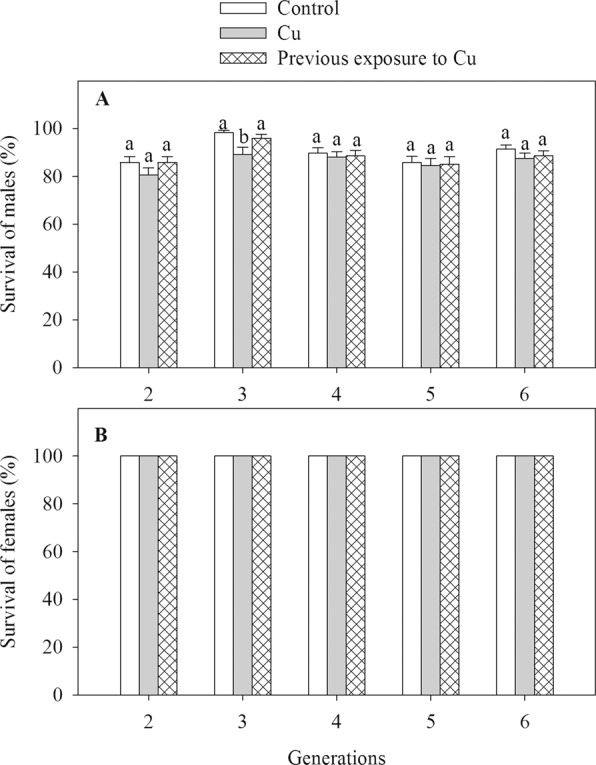
Survival of males (**A**) and females (**B**) of *Pseudodiaptomus annandalei* F2–F6 generations as a function of Cu treatment. Data are visualized as mean + 1 SE. Different letters above the bars of the same generation indicate significant differences among control, Cu and pCu treatment (Duncan Posthoc tests).

Overall, Cu exposure reduced male survival compared to the control (main effect of Cu, F_2, 60_ = 5.61, *P* = 0.006, Fig. 3A), but this pattern was only significant for F3 generation while there was no statistical difference in survival between control and Cu treatments in F2, F4, F5, and F6 generations (Cu × Generation, F_8, 60_ = 2.16, *P* = 0.043). Further, survival did not differ between the control and pCu treatment. Across all Cu treatments, male survival did not differ among generations (Generation, F_4, 60_ = 1.52, *P* = 0.21). For females, no mortality was observed in any of the generations (Fig. 3B).

**Figure 4:**
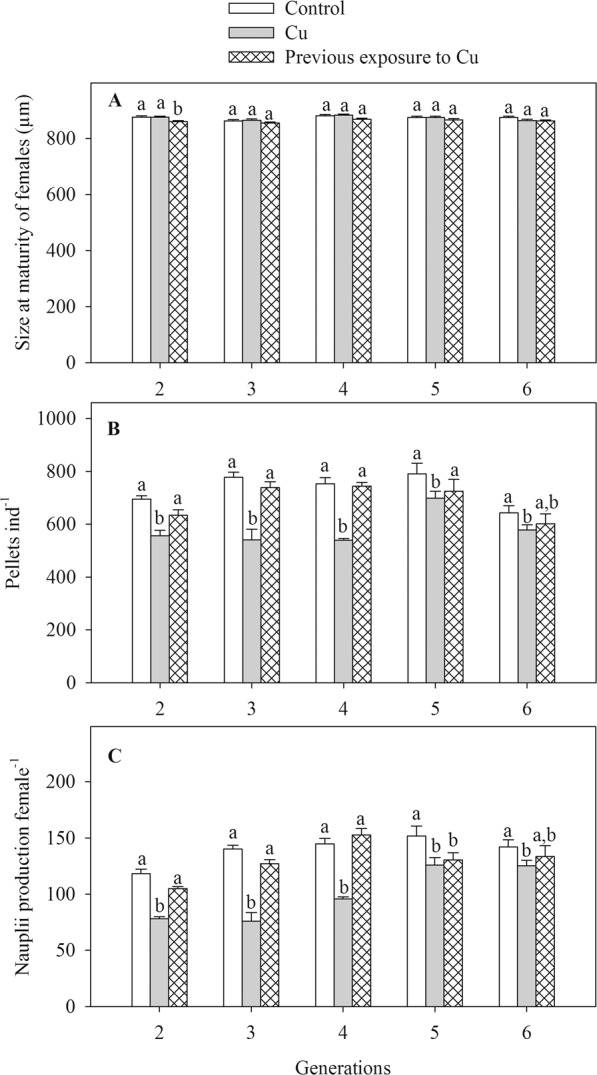
Size at maturity of females (**A**), faecal pellet (**B**) and nauplii production (**C**) of *Pseudodiaptomus annandalei* F2-F6 generations as a function of Cu treatment. Data are visualized as mean + 1 SE. Different letters above the bars of the same generation indicate significant differences among control, Cu and pCu treatment (Duncan Posthoc tests).

Female size was slightly reduced (~1%) in pCu treatment of F2 generation (Table 1, Fig. 4A). There was no effect of Cu or pCu on the size at maturity of females in F3-F6 generations. While there was a significant effect of generation on the size at maturity of females, the difference was small (~1%), and there was no clear trend of the changes in female size (Table 1, Fig. 4A). There was no interaction between Cu and generation on female size (Table 1).

**Table 1:** The results of general linear models testing for the effects of Cu exposure on the size at maturity of females, faecal pellet and nauplii production of *Pseudodiaptomus annandalei* from F2 to F6 generations. Significant *P* values are indicated in bold.

Effects	Size at maturity of females			Faecal pellet production			Nauplii production		
	df1, df2	F	*P*	df1, df2	F	*P*	df1, df2	F	*P*
Cu	2, 60	10.29	**<0.001**	2, 60	40.21	**<0.001**	2, 60	63.21	**<0.001**
Generation	4, 60	6.05	**<0.001**	4, 60	10.82	<**0.001**	4, 60	21.17	<**0.001**
Cu × Generation	8, 60	0.66	0.72	8, 60	3.21	**0.004**	8, 60	5.28	<**0.001**

Faecal pellet production was lower in the Cu treatment than in control, and this difference was more pronounced in F2-F4 generations, generating a main effect of Cu and the interaction of Cu × Generation (Table 1, Fig. 4B). In F2-F6 generations, faecal pellet production of pCu treatment was not statistically different from the control (Duncan Posthoc test, all *P*-values > 0.10). In F2-F4 generations, faecal pellet production of pCu copepods was higher than that of Cu-exposed copepods. Across Cu treatments, faecal pellet production varied from 10–20% among generations (Table 1).

Cu treatment and generations interacted to affect reproduction (Fig. 4C). From F2 to F3 generations, nauplii production was 34–46% lower in Cu-exposed females than in control females. Nauplii production of Cu-exposed females increased by 22 and 60% in F4 and F5 generations, respectively, compared to the F2 generation (Duncan Posthoc test, all *P*-values < 0.001). Nauplii production of F6 generation remained at the same level as in the F5 generation (Duncan Posthoc test, *P*-value > 0.05); both were about 83–88% compared to the nauplii production of control females. For the nauplii production of pCu females, it recovered to the control level in F2-F4, but it was as low as the Cu-exposed females in F5 and F6 generations.

Nauplii production covaried positively with fecal pellet production (F_1, 509_ = 643.84, *P* < 0.001, linear regression equation: Nauplii = 0.16 × faecals + 1.89, R^2^ = 0.57).

### F7 generation

**Figure 5:**
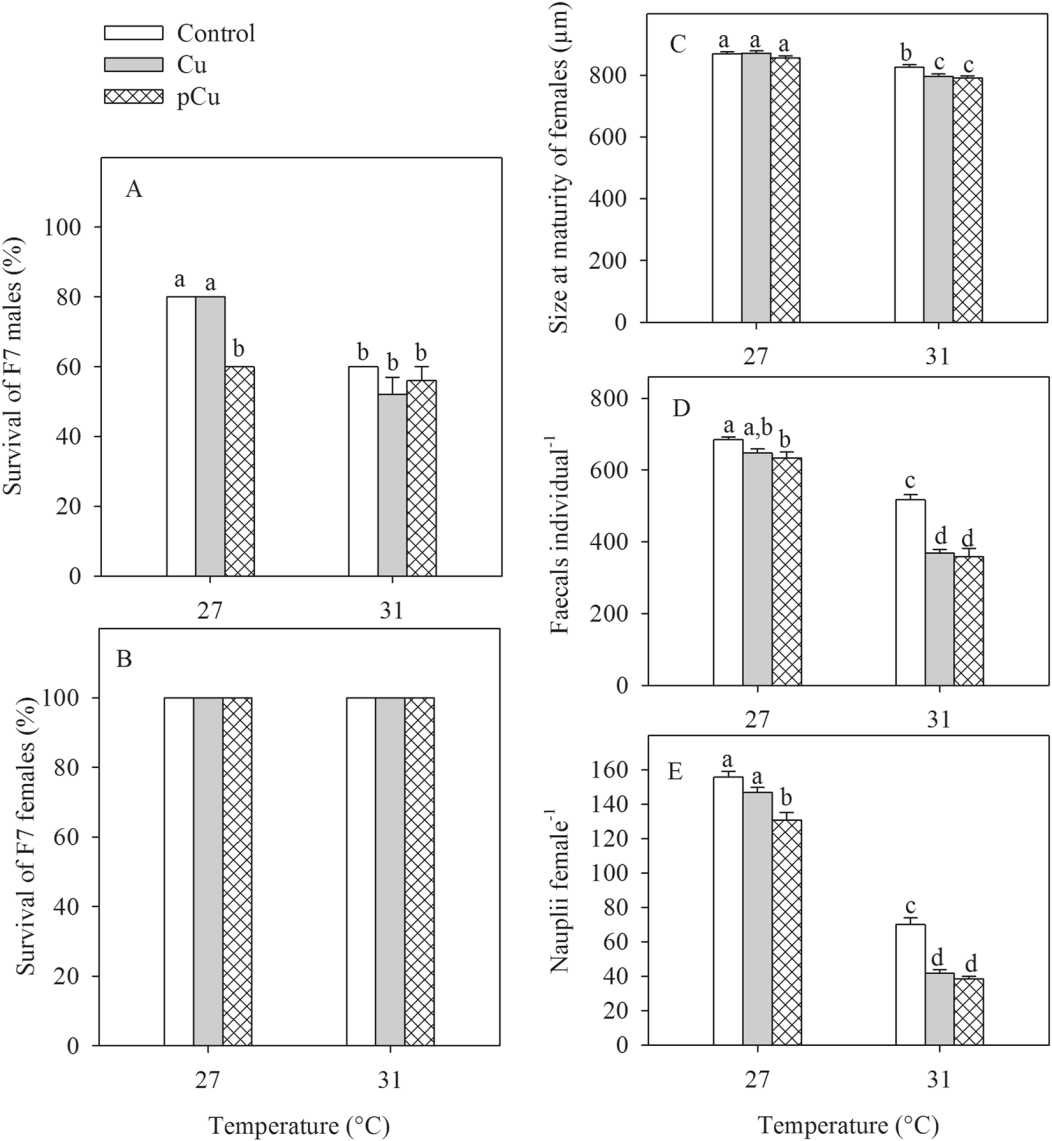
Survival of males (**A**) and females (**B**), faecal pellet (**C**) and nauplii production (**D**) of *Pseudodiaptomus annandalei* F7 generation as a function of Cu and temperature treatments. Data are visualized as mean + 1 SE. Different letters above the bars indicate significant differences among treatments (Duncan Posthoc tests).

For males, there was no difference in survival between control and Cu treatments at 27 °C, both had higher survival than in all other treatments (main effect of Cu). In the control and Cu treatment, the survival was considerably lower at 31 °C than at 27 °C (main effect of Temperature, F_1, 24_ = 67.60, *P* < 0.001) while temperature did not alter copepod survival in pCu, generating the Cu × Temperature interaction (F_2, 24_ = 11.2, *P* < 0.001, Fig. 5A). The survival of females was 100% in all treatments (Fig. 5B).

Size at maturity of females was reduced by 2 and 3% in Cu and pCu, respectively (main effect of Cu, Table 2, Fig. 5C). Across all three Cu treatments, size at maturity was 7% smaller at 31 °C compared to 27 °C (main effects of Temperature, Table 2, Fig. 5C). Generally, faecal pellet and nauplii productions were approximately 15 and 17%, and 17 and 25% lower in Cu and pCu treatments, respectively, compared to the control (main effects of Cu, Table 2, Fig. 5D,E). Across all Cu treatments, faecal pellet and nauplii production decreased by 37 and 65%, respectively, at 31 °C compared to 27 °C (main effect of temperature, Table 2, Fig. 5D,E). The Cu-induced reduction in faecal pellet and nauplii production was stronger at 31 °C than at 27 °C (interactions of Cu × Temperature, Table 2, Fig. 5E).

**Table 2:** The results of general linear models testing for the effects of Cu exposure and temperature on the size at maturity of females, faecal pellet and nauplii production of *Pseudodiaptomus annandalei* of the F7 generation. Significant *P* values are indicated in bold.

Effects	Size at maturity of females			Pellet production			Nauplii production		
	df1, df2	F	*P*	df1, df2	F	*P*	df1, df2	F	*P*
Cu	2, 24	5.89	**0.008**	2, 24	30.23	**<0.001**	2, 24	40.92	**<0.001**
Temperature	1, 24	109.49	**<0.001**	1, 24	400.81	<**0.001**	1, 24	1321.0	<**0.001**
Cu × Temperature	2, 24	2.83	0.079	2, 24	9.29	**0.0010**	2, 24	4.89	**0.016**

## Discussion

Copper is known to be highly toxic at elevated concentrations to marine organisms such as amphipods^27^, corals^28^, gastropods^29^, and worms^30^. In the present study, exposure to the ecologically relevant concentration of Cu did not affect survival and faecal pellet production but strongly reduced nauplii production. Nauplii production is a highly sensitive indicator of Cu toxicity in copepods^31^. We did not observe mortality of Cu-exposed copepods in the F1 generation, which is in agreement with the result of the range finding test (Supplementary Information S1). Cu exposure concentration of 15 µg L^−1^ is approximately 20 and 70 times lower than the reported 48h-LC_50_ of 310 µg Cu L^−1^ for *Tigriopus fulvus*^31^ and 48h-LC_50_ of 1024 µg Cu L^−1^ for *T. japonicus*^32^, respectively. Biandolino *et al*.^31^ showed that the number of nauplii per brood of *T. fulvus* was not affected by exposure to Cu concentrations of 15–60 µg L^−1^, suggesting that Cu did not affect the embryonic development and hatching success. In this study, nauplii production of *P. annandalei* was lower in the Cu treatment compared to the control, which may be the result of a higher number of aborted egg sacs and a lower number of broods produced per female in Cu-exposed copepods. In support, we found in a companion study that exposure to 15 µg Cu L^−1^ resulted in a four-time increase in the number of aborted egg sacs of *P. annandalei* compared to the control treatment (Doan X.N. Pham, Q.H., and Dinh K.V., in prep.). Other copepod species such as *T. fulvus* also shows a higher number of aborted egg sacs and a lower number of broods in Cu-exposed females^31^. Importantly, a lower nauplii production was observed while the faecal pellet production (a proxy for grazing rate^10,25,33^) was similar between Cu-exposed individuals and the control, indicating a higher energy expenditure on somatic maintenance and other physiological responses to deal with Cu. Exposure to Cu may trigger upregulation of metallothioneins^34^, cytochrome P450, heat shock proteins, ferritin, glutathione peroxidase (GPx) and glutathione S-transferase (GST). These play a role in xenobiotic metabolism, detoxification, antioxidant defense or stress response of marine copepods, such as *T. japonicus*^35,36^ and other marine species, such as *Mytilus coruscus*^37^.

While there was no lethal effect of Cu on F1 and on females of F2-F6 generations, males of F3 had lower survival in the Cu treatment, indicating a cumulative impact of Cu over generations like the cumulative effect of Cd on copepod *T. japonicus*^11^. This result is also in agreement with a generally higher contaminant sensitivity of males compared to females (no mortality observed for females). A higher contaminant sensitivity of males is common in copepod species^38–40^, which may be explained by the faster contaminant elimination in females via transfer to their eggs^11,40–42^.

As for survival, the reduced pellet production in F2-F4, but not in F1, may be a result of the accumulative effect of Cu exposure over generations. The lower grazing rate of marine copepods as a result of contaminant exposure has previously been observed^33,43,44^. The lower faecal pellet production suggests a lower energy intake, thereby less energy will be available for maintenance and reproduction. This may be further intensified by the metal-inhibited food digestion through depressing the activity of digestive enzymes, such as carbonxypeptidase B and chymotrypsin-like proteinase to break down ingested proteins from prey into amino acids, materials for the cellular metabolism^11^. Indeed, Cu-exposed females had a lower nauplii production. A positive correlation between faecal pellet and nauplii production was observed, which is similar to the finding in the copepod *Acartia tonsa* exposed to pyrene^10^, and this further supports our prediction of the metal-induced reduction in nauplii production mediated by grazing.

In our study, we found two important patterns of phenotypic plasticity and development of adaptation to Cu: (i) the transgenerational plasticity of F2-F4 in response to Cu exposure, and (ii) the development of adaptation to Cu in F5 and F6 generations. The F2-F4 generations of *P. annandalei* showed transgenerational plasticity in response to Cu exposure. Indeed, faecal pellet and nauplii production were lower in Cu-exposed copepods, but recovered in pCu-exposed copepods (no statistical difference between these two treatments), an indication of phenotypic plasticity^7,45,46^. Importantly, we found strong evidence of the development of adaptation to Cu of *P. annandalei* in F5 generation. The nauplii production of Cu-exposed females increased substantially compared to F2-F4 generations. Furthermore, nauplii production of pCu-exposed females did not return to the control level, and did not differ from the nauplii production of Cu-exposed females. Patterns of F5 generation were confirmed in the F6 generation. These results are in agreement with the prediction that the development of adaptation to contaminants (e.g. road salt - NaCl) in zooplankton species, such as *Daphnia pulex*, occurs within 5–10 generations. The cost of Cu adaptation was revealed when offspring from exposed parents was returned to the control, their nauplii production was not recovered like in F2-F4 generations. Interestingly, faecal pellet production of pCu-females was as high as that of the control, but their nauplii production was lower, indicating an energetic cost of adaptation.

It has been generally hypothesized that adaptation to contaminants may reduce the capacity of organisms to deal with climatic stressors, and *vice versa* (reviewed by Moe *et al*.^17^). Our study, using an evolutionary experiment, revealed that the development of Cu adaptation consistently resulted in stronger reductions in faecal pellet and nauplii production in *P. annandalei* under elevated temperature. Indeed, at 27 °C nauplii production was only reduced by 6% and 16% in Cu and pCu, respectively, yet at 31 °C it was reduced by 40% and 45% compared to the control. A similar pattern was observed for faecal pellet production with 5% and 7% reductions in Cu and pCu at 27 °C and 29% and 31% reduction at 31 °C compared to the control treatments at the respective temperatures. Nauplii production is a key trait for population growth. Our result provides strong empirical evidence for the “contaminant-induced climate change sensitivity” hypothesis (TICS)^17,23^. Metal contaminations are widespread in the coastal regions^4,5^ and are expected to become more severe in the near future from the rapid industrialization and urbanization^6^. Metal pollutions may cause higher vulnerability of marine species to environmental changes such as temperature (this study), ocean acidification^47^ and hypoxia^48,49^.

We also found a general and important pattern of the negative effect of temperature extremes alone on the performance of *P. annandalei*. Specifically, exposure to 31 °C resulted in lower survival (males), smaller size at maturity of females, lower faecal pellet and nauplii production. Lower male survival indicates that the temperature of 31 °C itself was lethal for *P. annandalei*. Such extreme temperatures are often observed in the shallow water in the tropical coastal regions of the South China Sea^25^ and are predicted to increase in frequency, duration, and severity in the coming years^50^. The temperature-induced reduction in size at maturity is a universal pattern that has been observed in various marine species from phytoplankton to fish^51–56^. The smaller size at maturity of female copepods typically show a lower grazing rate and fecundity^53,57^. Indeed, lower faecal pellet and nauplii production were observed at 31 °C than at 27 °C

Environmental pollution is a serious issue in tropical regions, particularly in the South China Sea^1,2,5,6^. Our study clearly shows that tropical zooplankton species can evolve in both transgenerational plasticity and development of metal adaptation as adaptive responses to exposure to ecologically relevant concentration of Cu. More generally, transgenerational plasticity and evolution of adaptations may occur widespread in nature as species have to cope with long-term changes in environmental factors such as the gradual increase in temperature^58,59^ and ocean acidification^60,61^. We revealed that the evolution of metal adaptation comes at the cost of reducing the capacity to deal with additional stressors; here we examined elevated temperature. This is important as increasing temperature, especially from marine heatwaves in this region^62^ is predicted to seriously affect the structures, function, and ecological services of the coastal ecosystems^63^. Detangling the transgenerational plasticity and evolution of adaptations to one stressor (Cu) and the associated costs would be the point of departure for tackling more complex issues on how marine species may persist, thrive or collapse under multiple-stressor conditions^64^.

Finally, our study showed that the negative effects of Cu on the survival, grazing, and reproductive success of *P. annandalei*, a key coastal zooplankton species, was found at Cu concentrations 10 times lower than the safety level of the current Vietnamese regulations^65^. While the impacts of widespread contaminants, such as metals^4–6^, on coastal species of this region are relatively unknown and poorly studied, newly emerging pollutants, such as plastics, arise rapidly^2,66^. The South China Sea is identified to be particularly vulnerable to climate change^15^, and climate change may further interact with contaminants to exacerbate the combined impact^17,67^. Altogether, it challenges efforts of biodiversity protection and management in one of the most biologically diverse and productive ecosystems on Earth, where substantial biodiversity loss has been documented^6,68,69^.

## Materials and methods

### Study species

The calanoid copepod *Pseudodiaptomus annandalei* distributes abundantly in tropical coastal ecosystems in the South China Sea region^26,70^. They are important grazers on small plankton^71^ and prey for fish larvae and juveniles^26^. The development time of *P. annandalei* lasts from 9 to 11 days at a temperature range of 25–34 °C^15,72^. On average, a female produces 0.8 clutches per day (9 clutches or 18 egg sacs in 11 days^72^. Each female can carry two egg sacs at a time, and each egg sac contains 4–10 eggs^72^. More than 90% of the eggs hatch into nauplii within 24 h at a temperature range of 28–30 °C^24^.

Adult copepod *P. annandalei* (approximately 2,000 individuals) were collected from Cam Ranh Bay in October 2017. They were transferred to the Laboratory of Live Feed in Aquaculture, The National Centre, where they were cultured in two tanks (200 L) at 27 °C and salinity of 20 ppt with ambient light and photoperiod (12 h light: 12 h dark cycle). The culture density was not controlled but varied between 150–300 individuals L^−1^, giving a total of 30,000–50,000 individuals per tank, which is approximately 30–50 times higher than the recommended culture density of copepods to maintain genetic diversity^7,73^. They were fed *ad libitum* on the microalga *Thalassiosira pseudonana*. To examine the performance of the F0 generation, copepods were collected randomly from the two tanks and mixed. Five groups of 5 males and 5 egg-carrying females (F0) were used to measure faecal pellet and nauplii production for 7 days. The average fecundity and the feacal pellet production of F0 were 21 ± 6 nauplii female^−1^ day^−1^, and 116 ± 15 pellets individual^−1^ day^−1^ (mean ± SD, n = 5).

### Microalgal culture

The diatom *Thalassiosira pseudonana* was cultivated in quartz glass bottles (volume = 3 L) in sterilized seawater at salinity 24 ppt enriched with f/2 media^74^. The cultivation system was placed in an air-conditioned room controlled at 25 ± 1 °C and continuous light. When the cultures reached a density of 12–16 million cells mL^−1^, algae were harvested to feed copepods. The harvested microalgae were then diluted by clean seawater to the designated food concentration of approximately 800 µg C L^−1^ for the copepods.

### Range finding tests

Prior to the evolutionary experiment, *P. annandalei* were exposed to one of five Cu^2+^ (hereafter Cu) concentrations: 0, 5, 15, 30, 60 and 120 µg Cu L^−1^ for 24 h. The survival, faecal pellet and nauplii production were quantified. Males had lower survival at Cu concentrations of 60–120 µg Cu L^−1^ (Fig. S1A, Supplementary Information S1). Survival of females was 100% in all Cu concentrations (Fig. S1B, Supplementary Information S1). The lowest Cu concentrations which resulted in reductions of faecal pellet and nauplli production were 15 and 30 µg Cu L^−1^, respectively (Fig. S1C,D, Supplementary Information S1). We selected an exposure concentration of 15 µg L^−1^ for the evolutionary experiment as this was the lowest concentration, which caused a significant negative effect on *P. annandalei* (i.e., the LOEC). This is ecologically relevant as the Cu concentrations >60 µg L^−1^ have recently reported for Vietnamese coastal water^5^, and approximately an order of magnitude below the safety level based on the National Regulations for Marine and coastal water quality (QCVN-10-MT-2015-BTNMT^65^).

To test whether the exposure concentration of Cu (15 µg Cu L^−1^) affect *T. pseudonana*, algae (180,000–200,000 cells mL^−1^) were exposed to either control (i.e., no Cu added) or Cu treatment (15 µg Cu L^−1^; five replicates per treatment) for 24 h. The initial and final algal densities were estimated by counting the number of cells in 1 mL-Lugol fixed samples. The final density of *T. pseudonana* were 184,000 ± 4,000 and 172,000 ± 4,899 cell mL^−1^ and did not statistically differ between Cu and the control treatment (ANOVA F_1, 8_ = 3.6, *P* = 0.09).

### Experimental design and setup

To test the how copepods may evolve in transgenerational plasticity and development of tolerance to metals (hypothesis 1), we exposed *P. annandalei* to Cu (0 vs 15 µg L^−1^) and tracked the changes in four key fitness-related traits: survival, size at maturity, grazing (faecal pellet production) and reproductive success (nauplii production). All parameters were determined in each generation until the development of Cu adaptation revealed by an improved performance (i.e., increased survival, faecal pellet and nauplii production) of copepods in Cu treatment, and that the performance of pCu copepods did not differ from those in the Cu treatment. The evolution of Cu adaptation appeared in F5 generation, which was then confirmed in F6 generation. From F2 to F6, the Cu treatment was split into two treatments: one group was continuously exposed to the same Cu concentration (15 µg L^−1^; Cu treatment) as in the previous generation(s), and the other group was returned to control conditions (i.e., Cu-free seawater; pCu treatment)^7^ (Fig. 1). This experimental design allows detangling whether, and when, the response of copepods were a result of phenotypic plasticity or the development of metal adaptation^7^. To further test whether the increased tolerance to metals may come at the cost of reducing the tolerance to another stressor (hypothesis 2), we used offspring produced by the F6 generation in an experimental setting crossing three Cu treatments (0, Cu and pCu) and two temperatures (27 and 31 °C) (Fig. 1). The later temperature simulated a 4 °C increase in mean temperature by 2100 due to global warming, scenario RCP 8.3 – “business as usual”^75^, and has often been recorded in the coastal water of the South China Sea^25^.

To prepare the exposure solution, CuSO_4_*5H_2_O (purity >99%, Merck, Germany) was dissolved in MQ-water. The exposure solution of 15 µg Cu L^−1^ was obtained by diluting the stock solution with clean seawater (20 ppt salinity).

To start the experiment, 200 females (F0) carrying two egg sacs were collected from the culture, they were placed in a 5-L glass bottle at room temperature of 27 °C and fed *ad libitum* with *T. pseudonana*. All nauplii were collected after 30h^15^ and ca. 150 nauplii were randomly assigned to each of 10 1-L bottles (five control and five Cu bottles) to start the F1 generation. The exposure solutions and algal food were renewed daily. When copepods developed into adults, three steps were performed:

(i) Five males and five females carrying two egg sacs were collected from each of the experimental bottles and transferred to another 1-L bottle of the same treatment to quantify the faecal pellet and nauplii production for 7 days. Each treatment had five replicates. Faecal pellets and nauplii were daily collected by filtering the water content in each bottle through a filter (mesh size = 30 μm). The content was poured into a plastic Petri dish, carefully rinsed and fixed in Lugol (4% final concentration). The number of faecal pellets and nauplii was counted under a microscope (SZ40, Olympus, Japan). The faecal pellet production was the cumulated faecal pellets produced by an individual over 7 days of the observation period. Likewise, the nauplii production was as the cumulated nauplii female^−1^ in 7 days. We checked mortality daily while refreshing the medium.
(ii) Ten females carrying two egg sacs were collected from each of the five 1-L control bottles and two groups of 10 females carrying two egg sacs were collected from each of five Cu-treatment bottles to start five control, five Cu, and five pCu bottles, respectively. All females produced nauplii for 30 h, then ~150 nauplii from each bottle were used to start the F2 generation (5 controls, 5 Cu and 5 pCu treatments). Other experimental conditions (food, temperature, salinity) were similar to the F1 generation. F3-F6 generations were started in the same way. For the F7 generation, each Cu treatment (control, Cu and pCu) was crossed with one of two temperatures (27 or 31 °C), resulting 6 treatments × 5 replicates = 30 experimental units (1-L bottles).
(iii) After producing nauplii for 30 h, 10 females from each group in step ii were fixed in Lugol (final concentration of 4%) and the prosome length (µm) was measured as the size at maturity. The average size at maturity of females from each 1-L experimental bottle was used for statistical analyses.

### Statistical analyses

One-way ANOVAs were used to test for the effects of Cu exposure on survival, size of females at maturity, faecal pellet and nauplii production of the F1 generation. For F2-F6 generations, we ran general linear models (GLMs) to test for the effects of Cu and pCu on the survival, size at maturity of females, faecal pellet and nauplii production across five generations. In these models, Cu treatment and generation were included as fixed factors. For all GLMs, we tested the assumption of normality of the error distributions with Shapiro-Wilk tests and the homogeneity of variances with Levene’s tests^76^. Survival, size at maturity of F7 females were were log(x + 1)-transformed to meet GLM model assumptions^77,78^. Following the ANOVAs and GLMs, we used Duncan posthoc tests for multiple comparisons, particularly among control, Cu and pCu treatmemts. Statistical differences were considered significant if P < 0.05. All statistical analyses were done in Statistica 12 (StatSoft Inc., Tulsa, OK, United States). Data are presented in the figures as the means + SEs.

### Data deposition

Data for this study are available at the Dryad Digital Repository when the manuscript is accepted for publication.

## Supplementary information

Supplementary Information.
