# Supplementary material for: Development of metal adaptation in a tropical marine zooplankton
Source: Sci Rep. 2020 Jun 23;10:10212. doi: 10.1038/s41598-020-67096-1 (PMC7311422; doi:10.1038/s41598-020-67096-1)
Supplement: Supplementary file 1 — Supplementary Information. [file 41598_2020_67096_MOESM1_ESM.docx]

Development of metal adaptation in a tropical marine zooplankton

Khuong V. Dinh^1,2,3,^*, Hanh T. Dinh^4^, Hong T. Pham^5^, Henriette Selck^2^, Kiem N. Truong^6,*^

^1^ School of Biological Sciences, Washington State University, Pullman, WA

^2^ Department of Science and Environment, Roskilde University, Universitetsvej 1, 4000 Roskilde, Denmark

^3^ Cam Ranh Centre for Tropical Marine Research and Aquaculture, Institute of Aquaculture, Nha Trang University, No 2 Nguyen Dinh Chieu Street, Nha Trang City, Vietnam

^4^ Northern National Broodstock Center for Mariculture, Research Institute for Aquaculture No 1, Xuan Dam Commune, Cat Ba, Hai Phong, Vietnam

^5^ Department of Environmental Engineering, Thuyloi University, 175 Tay Son, Dong Da, Hanoi, Vietnam

^6^ Department of Ecology, University of Science, Vietnam National University, Hanoi, 334 Nguyen Trai, Thanh Xuan, Ha Noi, Vietnam

*Corresponding authors: Khuong V. Dinh ([khuong.dinh@wsu.edu](mailto:khuong.dinh@wsu.edu)) and Kiem N. Truong ([kiemtn@vnu.edu.vn](mailto:kiemtn@vnu.edu.vn))

Telephone: +1 (509)-339-4903

**Supplementary information S1: Range finding test**

Copepod *Pseudodiaptomus annandalei* were exposed to one of five Cu concentrations: 0, 5, 15, 30, 60 and 120 µg L^-1^ for 24h. Each treatment had five replicates that were 1-L glass bottles; each contained 5 males and 5 females carrying two egg sacs. Copepods were fed *ad libitum* with *T. pseudonana*. The test was conducted at room temperature of 27°C. The survival, faecal pellet and nauplii production were quantified using a stereo microscope (SZ40, Olympus, Japan). Prior to analyses of variances (ANOVAs), data were log (x+1)-transformed to meet ANOVA assumptions.

Exposure to Cu reduced survival of males (main effect of Cu, F_5, 24_ = 13.72, *P* < 0.001). Duncan posthoc tests showed that survival of males was statistically lower at Cu concentration of 60 and 120 males than in the control (Duncan posthoc tests, *P*-values = 0.012 and 0.024, respectively, Fig. S1A). Survival of females was 100% in all Cu concentrations (Fig. S1B). Faecal pellet and nauplli production were lower at the Cu concentration of 15-120 and 30-120 µg L^-1^, respectively, than the control (ANOVA, faecal pellet: F_5, 24_ = 17.01, *P* < 0.001 and nauplii production: F_5, 24_ = 72.22, *P* < 0.001, Fig. S1C,D).


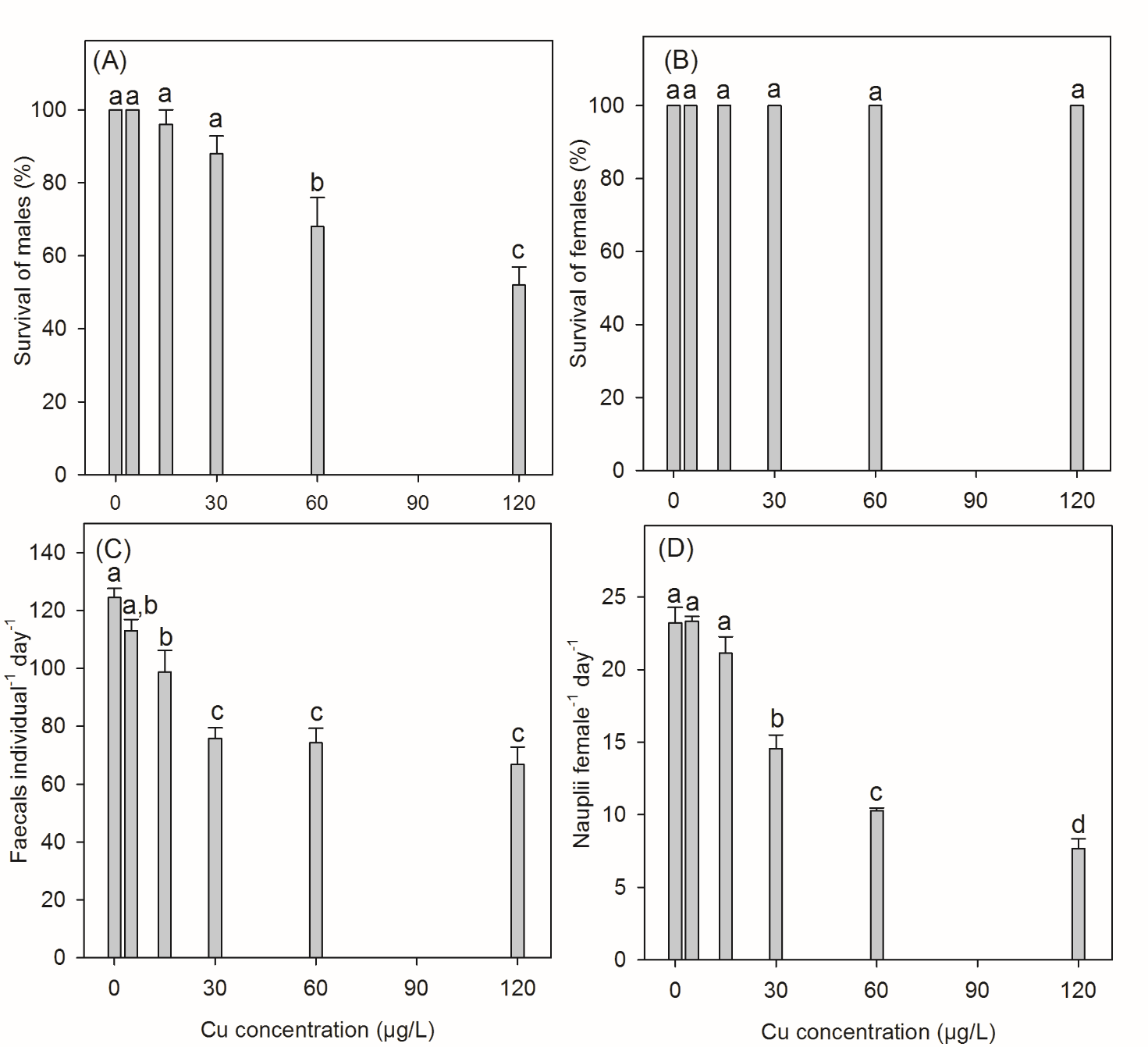


**Figure S1**. Survival of males (A), females (B), faecal pellet (C) and nauplii production (D) of the tropical copepod *Pseudodiaptomus annandalei* as a function of Cu exposure. Data are means + 1 SE. Letters above the bars indicate statistical significances (*P* < 0005, Duncan posthoc test).
